# High Redox Status as the Basis for Heavy Metal Tolerance of *Sesuvium portulacastrum* L. Inhabiting Contaminated Soil in Jeddah, Saudi Arabia

**DOI:** 10.3390/antiox11010019

**Published:** 2021-12-22

**Authors:** Emad A. Alsherif, Turki M. Al-Shaikh, Omar Almaghrabi, Hamada AbdElgawad

**Affiliations:** 1 Biology Department, College of Science and Arts at Khulis, University of Jeddah, Jeddah 21959, Saudi Arabia; tmalshaikh@uj.edu.sa; 2 Department of Biology, College of Science, University of Jeddah, Jeddah 21959, Saudi Arabia; oalmaghrabi@uj.edu.sa; 3 Integrated Molecular Plant Physiology Research, Department of Biology, University of Antwerp, 2020 Antwerp, Belgium; hamada.abdelgawad@uantwerpen.be; 4 Department of Botany and Microbiology, Faculty of Science, Beni-Suef University, Beni Suef 62511, Egypt

**Keywords:** phytoremediation, pollution, vegetation, bioindicator, biodiversity

## Abstract

Because sewage sludge is contaminated with heavy metals, its disposal in the soil may pose risks to the ecosystem. Thus, heavy metal remediation is necessary to reduce the associated risks. The goal of this research is to introduce a heavy metal resistant species and to assess its phytoremediation, oxidative damage markers and stress tolerance mechanisms. To this end, field research was done to compare the vegetation of polluted sites to that of a healthy site. We found 42 plant species identified in the study, *Sesuvium portulacastrum* L. was chosen because of its high relative density (10.3) and maximum frequency (100 percent) in the most contaminated areas. In particular, *S. portulacastrum* plants were characterized by strong Cu, Ni, and As uptake. At the organ level, to control growth reduction and oxidase damage, particularly in roots, increased detoxification (e.g., metallothionein, phytochelatins) and antioxidants mechanisms (e.g., tocopherols, glutathione, peroxidases). On the other hand, flavonoids content and the activity of glutathione-S transferase, glutathione reductase and dehydroascorbate reductase were increased manly in the shoots. These biochemical markers can be applied to select tolerance plant species grown under complex heavy metal contamination. Our findings also introduced *S. portulacastrum* to reduce soil contamination0associated risks, making the land resource available for agricultural production.

## 1. Introduction

Heavy metals discharged into the environment as a result of human activities can create major pollution [1]. Sludge dumping and large-scale effluent water generation resulted in heavy metal contamination in soil [2]. Because of their long-term persistence in the ecosystem, hazardous heavy metals released into the soil are especially worrying. Heavy metals have a high rate of transfer from soil to forage and food crops; consequently, its presence in the soil in high concentration will induce their accumulation in plants [3]. As a result, they disrupt plant metabolism and growth; also, their accumulation in plants poses a serious hazard to human and animal health as a result of food chain bio-magnification [3].

Soil pollution drivers (factors) can have a large impact on individual species, plant groupings, and ecosystems [4]. Previously, it was documented that environmental disturbances can be identified by looking at species diversity, annual species density, perennial species density, and vegetation cover. These ecological traits are essential for identifying prospective indicator species as well as monitoring pollution effects [5]. Furthermore, they contribute to a greater comprehension of the evolution of species that are both sensitive and tolerant in response to pollution [6]. As a result, numerous methods for removing hazardous heavy metals from polluted soil have recently emerged [7]. Bioremediation, which employs microbes or higher plants, is an environmentally beneficial process that successfully removes toxic from the environment [8,9,10,11]. Phytoremediation by higher plants, in particular, may be carried out under a variety of environmental situations [12,13].

Heavy metal phytoremediation causes changes in metabolic homeostasis in tolerant plants, such as an increase in demand for secondary active metabolites to cope with the heavy metals toxicity [14]. In response to sublethal doses of various heavy metals, our prior investigations revealed obvious variations in detoxification and antioxidants between sensitive and resistant plants. Despite the fact that heavy metal contamination has a negative impact on soil health, it has a huge genetic resource of stress-tolerant plants [14]. Metallochaperones and chelators, which sequester additional heavy metals into vacuoles, are part of a stress-tolerant plant’s detoxifying mechanism [15,16].

To combat oxidative stress caused by heavy metal stress, tolerant plants activate certain antioxidant components in their arsenal [17]. Although the relation between the induced antioxidant defense system and the tolerance to heavy metal stress is well known, it is hardly studied succulent plants such as *Sesuvium portulacastrum.* Moreover, very little is known about the specificity of stress biochemical markers and their proper practical application to select stress-tolerant plants grown under complex heavy metal contamination [18].

*S. portulacastrum* (Aizoaceae), a spreading perennial herb that may grow up to 30 cm tall and has thick, smooth stems that can exceed one meter in length, is one of these plants that occupy polluted places and endure pollution and hard weather. It may be found in coastal limestone and sandstone, sandy clay, and salt marshes all over the world. Based on total arsenic (As) accumulation, bioaccumulation factor, and acknowledged biomass production capacity, *S. portulacastrum* has been proposed as a viable contender for use in arsenic removal and land re-vegetation/reclamation operations in As-contaminated sites across the world [19].

This research looked at the effects of heavy metal poisoning on plant biodiversity and vegetation in a polluted region of Jedda. The plant community in this location was screened, and a high stress-tolerant species, *S. portulacastrum*, was selected for further growth and biochemical analyses. This also provided a set of biochemical markers that can be used to screen for stress tolerance plants grown under in filed under complex heavy metal contamination. Overall, this research also advances our understanding of the stress mitigation mechanisms and biochemical flexibility of distinct organs *S. portulacastrum*, as well as whether they are connected to organ type.

## 2. Material and Methods

### 2.1. Study Area Description

**Figure 1:**
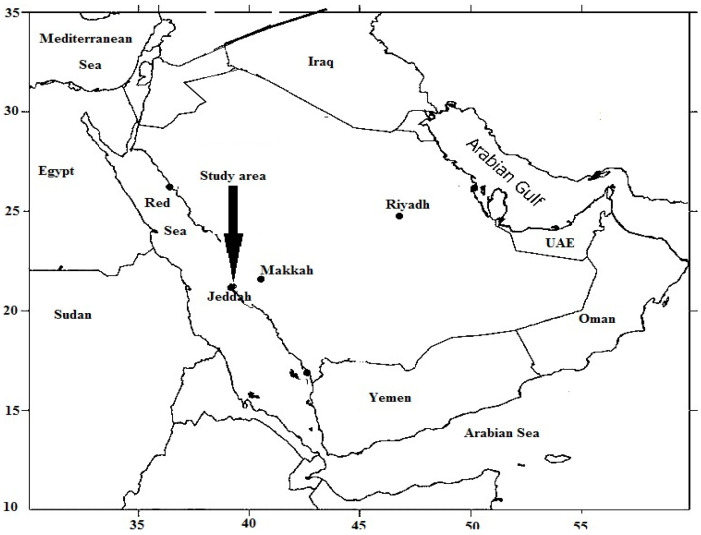
Map showing the study site location.

The study was completed around a man-made sewage dumping lake located east of Jeddah City (Figure 1), Saudi Arabia, in a low-lying floodplain of the hilly terrain (21°35′11.90″ N 39°19′29.26″ E). It was used as a sewage dumping lake with no treatment measures. Approximately 5 × 10^4^ m^3^/day of effluent was transported into Al-Musk Lake via tanker. It is located at around 150 m above sea level in the Bani Malek valley watershed. The climate in the study region is hot with rare rainfall (Supplementary Table S1).

### 2.2. Sites Localization

Five unique sites around the sewage sludge lake were chosen for the soil and vegetation inquiry, each with a 25,000 square meter area. The sites were chosen based on slope, soil type, depth, pH, and area size, as well as their closeness to the sewage dumping lake. There was no land usage and no disturbances such as livestock, roads, or other pollution sources at any of the study sites. The first site (S1) is 50 m from the sewage dumping lake, the second is 100 m, the third is 500 m, the fourth is one kilometer, and the nonpolluted control zone is five kilometers away (Sc).

### 2.3. Field Surveys

Vegetation surveys were carried out using the method of quadrats’ points [20]. Plant species compositions were obtained by randomly planting one square meter quadrat at ten different sites on each site. Species number, density, and vegetative cover were all detected [21]. With the use of standard flora reference books, plant species within each quadrat were collected and identified [22,23,24,25]. Life form categories were constructed according to Raunkiaer’s guidelines [26]. When a taxon contains a variety of life forms, the most representative taxon was chosen; fluctuations in the life form in the field were ignored. The fundamental method and terminology of Zohary [27] for the Saharo-Arabian and Sudanian areas will be used to avoid the diverse notions of chorological units among writers, which has resulted in several designations for Saudi Arabia’s two principal regions.′

### 2.4. Floristic Diversity Analysis

To characterize and compare species diversity among the examined sites, Shannon’s diversity index (H) and the Pielou evenness index (Ep) were detected [28]. The following is how Shannon’s diversity index is calculated:

H′= −Σpi ln pi: pi = ni/N and ln indicate the natural logarithm.

Pielou evenness index is given as:

Ep = H′/ln S: S denotes the diversity of species. The similarity coefficient of Jaccard was utilized to assess the gradient of diversity changes between the five examined sites according to the formula below:

Cj = [a/(b + c + a)] × 100

where a denotes the total number of species discovered at both locations: b the number of different species discovered only at the first site, while the number of species is denoted by the letter c. discovered exclusively at the second site.

Relative density (RD) is a measure of a species’ overall number of individuals in proportion to all other species’ individuals, determined as:

Relative Density (RD%) = (Individuals number of the specific species divided by the total number of all individuals for all recorded species) × 100.

The distribution of a species, expressed as a percentage of occurrence, is known as frequency (F).

Frequency (F%) = (Total number of quadrates studied/number of quadrates where the species occurred) × 100

### 2.5. Collection of S. portulacastrum

Five samples were obtained from the rhizosphere at each location, all of which were of the same age, and placed in airtight polyethylene zipper bags before being transferred to the laboratory. Roots and shoots were separated and fresh weights collected, then air-dried at room temperature, weighed, and stored in appropriate containers until chemical analysis. The amounts of Cd, As, Hg, Al, Fe, Cu, V, Cr, Ni, Co, Pb and Zn, were established through the digestion of ground plant material in a concentrated acid combination reported by Violante et al. [29].

### 2.6. Biological Indices

Biological indices were used to analyze the interaction between plant and mineral, also the metal absorption capacity of various plant species [30]. Metals’ BCF (also known as biological concentration factor) is determined by dividing the metal concentration in the plant’s root by the soil metal content. TF is the ratio of metal content in the shoot to metal content in the root, known as the translocation factor.

### 2.7. Heavy Metal and Mineral Content in Soil and Plant Organs

To eliminate any apoplastic collected metal ions, plant leaves and roots were rinsed with deionized water. Heavy metals and minerals were determined after 150 mg of the dry weight of plants and 3 g of soil were digested in HNO_3_/H_2_O (5:1) and determined (mass spectrometry, ICP-MS). After that, standards in 1% (*v*/*v*) HNO_3_ were prepared [31]. Heavy metal contents were written as μg/g DW of soil. To eliminate any apoplastic collected metal ions, Milli-Q water was used to wash the roots and leaves tissues. Heavy metals and minerals were detected after 150 mg of dry weight of plants and 3 g of soil were digested in HNO_3_/H_2_O (5:1) and calculated (mass spectrometry, ICP-MS). After that, standards were made in 1% (*v*/*v*) nitric acid [31]. The heavy metal concentration of the soil was measured in g/g DW.

### 2.8. Quantification of Organic Acids

Organic acid (Citric acid) was taken out (Butylated hydroxyanisole in 0.1 percent phosphoric acid). The content was analyzed by using HPLC methods (LaChrom L-7455 diode array, (Merck, Darmstadt, Germany) De Sousa et al. [32]. Ribitol was used as an internal standard.

### 2.9. Photosynthesis and Photorespiration Related Parameters

In homogenized shoots, the amounts of chlorophyll a and b, as well as carotenoids, were measured in acetone [33]. The photorespiration-related essential enzymes including GO (Glycolate oxidase) and HPR (hydroxy pyruvate reductase) activities were assessed according to Feierabend and Beevers [34] and Schwitzguebel and Siegenthaler 1984, respectively. Moreover, the glycine/serine ratio known as an indicator of photorespiration) [35]. A Waters Acquity UPLC-TQD system (Waters, Milford, MA, USA) with a BEH amide 2.150 column was used to quantify glycine and serine.

### 2.10. Quantification of Oxidative Damage Markers

The quantities of H_2_O_2_ were determined by using the FOX1 technique to monitor the Fe^3+-^xylenol orange complex at 595 [36]. The amount of lipid peroxidation was evaluated using the thiobarbituric acid-malondialdehyde (TBA-MDA) reagent after homogenized plant tissues were extracted in 80 percent ethanol [37]. The content was expressed as nmol. g1 fresh weight and different absorbances (440, 532, and 600 nm) were recorded. Protein carbonyls were detected as oxidative damage indicators by Cayman Chemical’s (Ann Arbor, MI, USA) Protein Carbonyl Colorimetric Assay Kit [38].

### 2.11. Quantification of Antioxidant Parameters

Total antioxidant capacity (FRAP) and antioxidants (phenolics and flavonoids) were extracted in 80% ethanol. After centrifugation (14,000× *g*, 4 °C, 25 min), the FRAP assay (0.3 M acetate buffer (pH 3.6), TPTZ (0.01 mM) in HCl (0.04 mM), and 0.02 M FeCl_3_.6H_2_O) were performed with Trolox (0 to 650 M) as a reference [39]. In the sample supernatant, polyphenols and flavonoids were assessed [40]. The flavonoid content was calculated using the modified aluminum chloride approach [41]. Ascorbate (AsA) and glutathione (GSH) were measured by HPLC analysis. After separation on a reversed phase of an HPLC column (Polaris C18-A (100 × 4.6 mm), particle size (3 m), and 42 °C, plant samples were extracted in meta-phosphoric acid (6%, *w*/*v*). ASC and GSH were detected by a diode array detector (DAD) [31]. Proteins were extracted using K-phosphate extraction buffer (50 mM and pH 7.0) containing PVPP (10% *w*/*v*), Triton X-100 (0.25 percent *v*/*v*), and PMSF for antioxidant enzyme activity (1 mM). The oxidation of pyrogallol at 430 nm (Kumar and Khan, 1982) and superoxide dismutase (SOD) enzyme activities, as well as the suppression of NBT reduction at 560 nm, were used to determine the activity of peroxidase (POX) [41]. Spectrophotometric analysis of dehydro-ASC reductase (DHAR), GSH reductase (GR), ascorbate peroxidase (APX), and monodehydro-ASC reductase (MDHAR) was performed using the Murshed et al. [42] technique with 0.05 M MES/KOH. The rate of breakdown of H_2_O_2_ at 240 nm was used to evaluate catalase (CAT) activity (Aebi, 1984). The activity of glutathione peroxidase (GPX) was determined by measuring the reduction of NADPH at 340 nm [43]. The Lowry method was used to determine the total soluble protein content [44].

### 2.12. Quantification of Detoxification Related Parameters

GSH-S-transferase was extracted using 0.5 mM CDNB and 1 mM GSH in K-phosphate buffer (50 mM, pH 7.0). Mozer et al. calculated the activity [45]. According to Diopan et al., the content of metallothionein (MTC) was determined electrochemically (pulse voltammetry Brdicka reaction. After combining with Ellman’s reagent, the amount of phytochelatins (total thiols-non-protein) was extracted (5 percent sulfosalicylic acid) and spectrophotometry measured at 412 nm [46].

### 2.13. Statistical Analysis

A one-way ANOVA (Tukey test (*p* < 0.05), SPSS 20.0 software, (SPSS 22.0 for Windows; SPSS Inc., Chicago, IL, USA) was applied to estimate if there are significant responses in response to the treatments impact on root and shoot (*n* = 4). PCA was performed (Origin Lab 9, Corp., Northampton, MA, USA) to identify the variability of the results.

## 3. Results

### 3.1. Effect of Sewage Pollution on Floristic Composition

**Figure 2:**
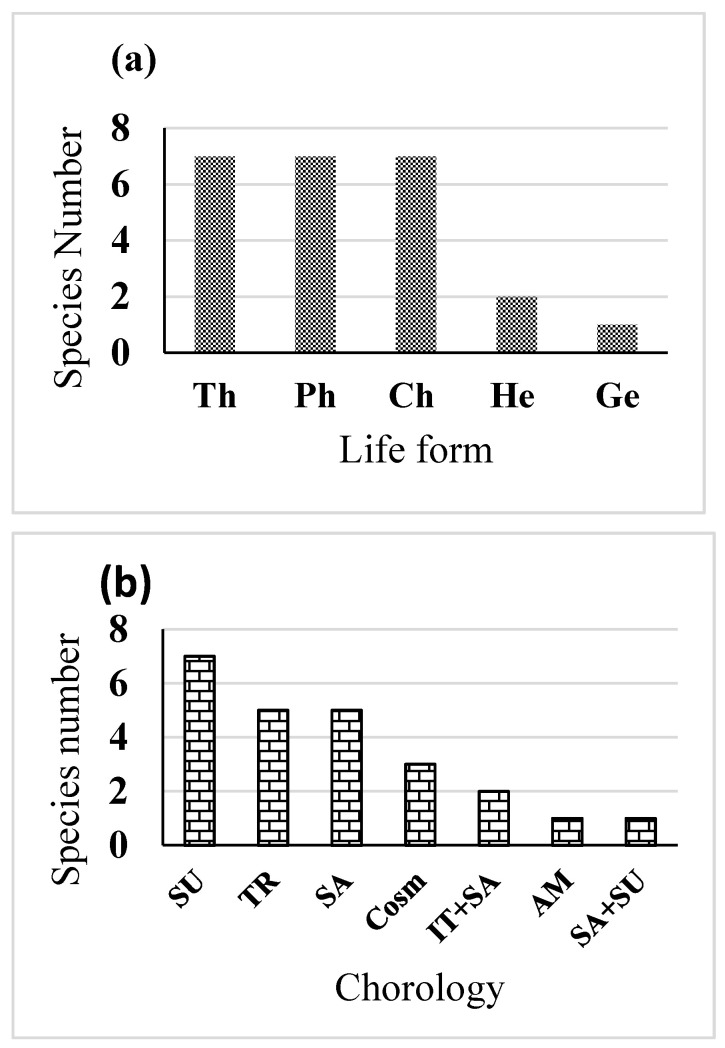
(**a**) Life form: Ph, phanerophytes; Ch, chamaephytes; G, geophytes; He, hemi-cryptophytes and Th, therophytes and (**b**): Chorology: COSM, cosmopolitan AM, American; IT, Irano-Turanian; TR, Tropical. Mediterranean; SA, Saharo-Arabian; SU, Sudano-Zambezian and TR, Tropical of the recorded species.

To investigate the degree of heavy metal contamination in the soils of each site, several heavy metal concentrations in the rhizosphere soils of *S*. *portulacastrum* plants were measured. Depending on the concentrations of these heavy metals, we ranked the contaminated soils into 5 levels, from Site 1 (control) to site 5 (the highest contamination). Twenty-four plant species belonging to 23 genera and 13 families were recorded (Supplementary Table S2). The major plant families present in the area in question were Poaceae (5 species) followed by Fabaceae and Aizoaceae (3 species for each). Concerning the life forms recorded in the present study, each Therophytes, Chaemophytes, and Phanerophytes recorded 29%, while the least life form class (1%) was Geophytes (Figure 2a). In the most polluted site, *S. portulacastrum* L. had the highest frequency of 100% with 10.3 relative density. *P. juliflora* (Sw.) DC. had a relative density of 3.5 and a frequency of 90%, followed by *L. fusca* (L.) Kunth (RD = 8.5, F = 61%). The chorological characteristics of the recorded species showed that Sudano-Zambezian recorded the highest number (29%) followed by Saharo-Arabian and Irano-Turanian elements, (20%) (Figure 2b). It was observed that the greater the distance between the site and the sewage dumping lake, the greater the vegetation cover (Table 1).

A clear variation in the vegetation cover between site 1 (control site) and the other 4 sites (*p* = 0.03) was noticed. The vegetation cover (Table 1) was decreased by 50% in the sewage dumping lake vicinity (site 5), compared to the control (S1). The species number and diversity measurement of the target community are reported in Table 1. The pollution had a significant impact on the plant richness in the area closest to the sewage lake (S5), where plant richness was reduced by 58% as compared to the non-polluted region (S1). Furthermore, the change of this characteristic across sites was extremely significant (*p* = 0.05). Table 1 shows that the Shannon–Weiner Index (H’) reflects the ecosystem’s health, with the control site having a higher H′ value of 3.56 than the contaminated sites, which ranged between 2.01 to 3.02. The control site exhibited floristic heterogeneity in contrast to site 5. 

On the other hand, site 5 had more common species than the other sites. There was variability in species composition across site 5 and the control site, with 8% of common species. When comparing the results of pollution sites to the control site, the lower index, a decrease of about 30%, reported at the control site was due to the difference in species incidence. In the polluted sites (site 2–site 5), *S. portulacastrum*, *P. juliflora*, *E. colona*, and *L. fusca* had high frequencies, reaching 90%, 50% and 60%, respectively. The previous species also had high relative densities (RD), reaching 10.3, 8.9 and 9.1, respectively, suggesting their tolerance for heavy metal buildup by wastewater (Supplementary Table S1).

### 3.2. Heavy Metals Level in Contaminated Soils and Their Uptake by S. portulacastrum

Four out of 12 detected heavy metals, i.e., Pb, Co, Hg and Cd showed the highest levels. Compared to the control Site, the polluted sites showed gradual increases in the concentrations of eight metals. Table 2 shows that the content of the three metals, Pb, Co and Hg, represented 68% of the total heavy metals at the most contaminated site (S5). Moreover, Ni, Cu and Cr exhibited high concentrations. On the other hand, soils showed considerable levels of several essential and non-essential minerals, including N, Ca, K, and Mg, whereas their levels were not significantly affected by heavy metal accumulation in soil (Table 2). The accumulations were measured in both the plant shoots and the roots. Pb, Co, Hg, Cd, Ni, Cu and Cr levels were sharply increased in both organs of *S. portulacastrum* plants and to a greater extent in the roots of plants grown in contaminated soil at Site 5 (Table 3). Similar to their level in soil, the highest accumulation was recorded for Pb and Co and Hg more than 200 folds, for *S. portulacastrum* shoots and roots at site 5 compared to Site 1 (Table 3). Compared to plants grown in control soil (site 1), plants grown in contaminated sites, particularly site 5, showed increased levels of other heavy metals such as Cu, Ni, and Cr. Consequently, altered plant mineral nutrition due to their competition with heavy metals was observed. Thus, the concentrations of essential plant nutrients such as N, K, Mg and Mn in both the shoots and roots of *S. portulacastrum* plants were evaluated in the present study to determine the state of plant nutrition (Table 3). Interestingly, heavy metal accumulation reduced K uptake by the root and shoot organs by 65% and 62%, respectively (Table 3). Nitrogen was reduced by 12% in both roots and shoots of *S. portulacastrum*, while the decrease in Mn was detected by 35% in both organs. Mg uptake was slightly reduced by 9% the shoots and roots of *S. portulacastrum* plants grown in the most contaminated soil, respectively (Table 3).

It has been observed that with the increase in the heavy metal concentration in the soil, the values of both BCF and TF increase, recording the highest values of both in the most polluted site (Table 4). The results indicated high content of Pb, Co, Hg and Cd in both roots and shoots, in addition to a high uptake (BCF), low translocation from root to shoot (TF). The highest biological concentration factor (BCF) was recorded by Cu, followed by Ni and As, while the lowest value was detected by Al. At the most contaminated site (5), it was interesting to find that the metals that recorded the highest concentrations in soil and plant organs of *S. portulacastrum* (Pb, Co, Cd) had lower BCF than many other elements such as Ni, Cu, and As. The translocation factor (TF) of all the studied metals recorded values less than one. The lowest TF was recorded by Cu, followed by those recoded by Pb and Zn. The translocation factor of metals (TF) in *S. portulacastrum* ranged between 0.20 and 0.74 at the most contaminated site. Cu recorded the lowest value, while Fe recorded the highest one. In the case of *S. portulacastrum* growing in healthy habitats (control site), the BCF ranged between 22.44 and 57 for the detected heavy metals, while the TF of metals ranged between 0.08 and 0.74.

### 3.3. Growth Responses to Soil Contamination with Heavy Metals

To evaluate *S. portulacastrum* responses to soil contamination, plant growth, fresh weight (FW), dry weight (DW), and pigment content were measured (Table 5). The result showed that soil contamination significantly induced growth reduction, particularly for the plant roots grown at Site 5 as compared to those grown at the control site (S1). We measured photosynthetic pigments (Table 5) to investigate the integration of heavy metal accumulation on photosynthetic related parameters and its relationship with the higher growth of *S. portulacastrum* plant. The increase was more pronounced (20%) for Cha as compared to Chb and Cha + Chb at site 5. The carotenoids were enhanced in response to heavy metal contamination, compared to those of control plants. Furthermore, this increase was stimulated in plants grown in the most contaminated soil at Site 5, recording an increase of more than 100%. *S. portulacastrum* has maintained growth in the most polluted site, although its growth showed a decrease in fresh and dry weights by 30% and 31%, respectively. We selected *S. portulacastrum,* which exhibited the highest frequencies and relative densities in polluted sites (Table 5).

### 3.4. ROS Production and Oxidative Damage

To improve our understanding of the downstream effects of heavy metal contamination on ROS levels and production, we investigated their effects on photorespiration, the main source of ROS (Table 6). In this context, the photorespiration-related enzymes glycolate oxidase (GOX), hydroxy-pyruvate reductase (HPR) and indicator (Gly/Ser ratio), as well as H_2_O_2_ accumulation in response to heavy metal accumulation were investigated. Plants grown in contaminated soil at sites 2–5 showed significant increases in H_2_O_2_ by 8%, 41%, 30% and 33% in shoot tissues and 11%, 29%, 40% and 63% in root tissues of *S. portulacastrum* plants, respectively, as compared to their corresponding control plants (Table 6). Consistent with the heavy metals-induced H_2_O_2_ accumulation, a significant increase was observed in the photorespiratory indicator Gly/Ser ratio and the GOX and HPR, mainly in plants grown in the most contaminated soil of site 5.

In more detail, exposing plants to heavy metal stress at sites 2, 4, and 5 increased GOX by 11%, 21%, and 158%, respectively, and the Gly/Ser ratio by 21% and 126%, respectively, at sites 4 and 5. The obtained results show that HPR was increased by 49% at the most contaminated site.

To investigate if heavy metal-induced mitigating oxidative responses in *S. portulacastrum* and if there were organ-specific responses, we quantified heavy metal induced malondialdehyde (MDA, used as a marker of ROS induced lipid peroxidation) and protein oxidation (PO) (Table 6). High heavy metal accumulation in site 3 and/or site 4 had a significant effect on oxidative stress markers in both organs of *S. portulacastrum* plants. Heavy metal contamination resulted in significant increases in MDA by 152% and 139%, and PO by 8% and 8.3% in the shoots and roots tissues, respectively, as compared to their corresponding control plants.

### 3.5. S. portulacastrum Showed Induced Defense System

**Figure 3:**
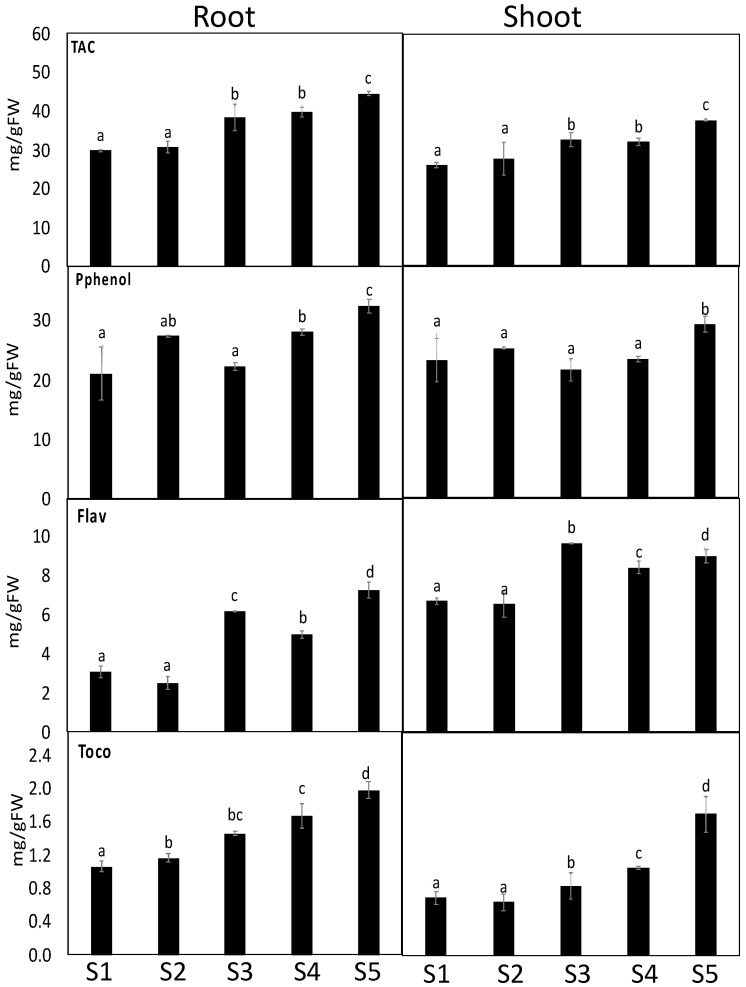
The impact of soil pollution on total antioxidant metabolites in the shoots and roots of *S. portulacastrum.* The changes in total antioxidant capacity (TAC, FRAP), polyphenols (Pphenol), flavonoids (Flav) and tocopherols (Toco) in *S. portulacastrum* grown in different control sites (site 5) and contaminated sites (sites 1–4). Data are mean values ± SD (*n* = 4). Different letters (a–d) indicate statistically significant difference between means of the same plant species at significance level at least (*p* ≤ 0.05).

**Figure 4:**
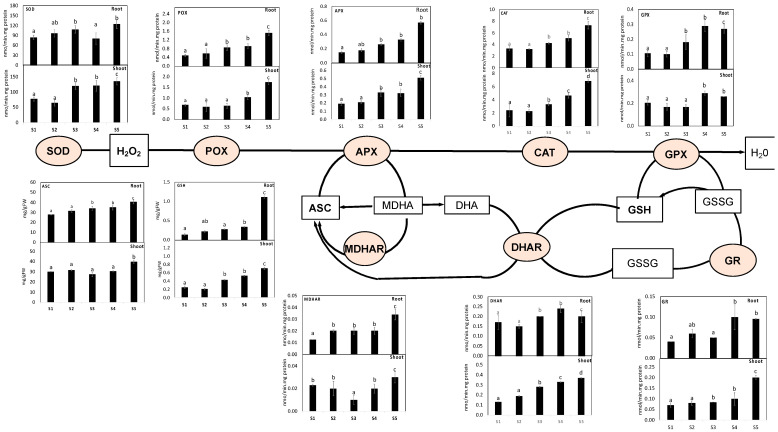
The impacts of soil pollution on ASC-GSH cycle-related metabolites and enzymes in the shoots and roots of *S. portulacastrum* strum grown in different control sites (site 5) and contaminated sites (site 1–4). Data are mean values ± SD (*n* = 4). Different letters indicate a statistically significant difference between means of the same plant species at a significance level at least (*p* ≤ 0.05).

Plant metabolism involves numerous oxidative reactions essential for cell viability under heavy metal toxicity, where plants use different antioxidant arsenals to combat heavy metal-induced oxidative stress. Out of the contaminated soil of the studied four sites, soil from Site 5 showed the highest impact on the antioxidant defense system. First, we measured the antioxidant capacity (FRAP) as well as several antioxidant metabolites. Our results revealed that plants grown in soil contaminated with heavy metals recorded significant increases in FRAP by 60% and 81% in the shoots and roots of *S. portulacastrum* grown in site 4, respectively. Similarly, the polyphenol and flavonoids, as the main contributors to FRAP changes, were increased by 27% and 139%, respectively, in *S. portulacastrum* root tissue (Figure 3). Changes in the lipid antioxidant (tocopherols) and ascorbate-glutathione (ASC-GSH) cycle-related metabolites and enzymes were measured (Figure 4). The roots of *S. portulacastrum* plants growing in the highest contaminated site exhibited significant increases in ASC (46%), while shoots and roots showed an increase in GSH (184% and 690%, respectively) and tocopherols (198% and 145%, respectively) levels as compared to their corresponding control plants (Figure 3 and Figure 4). Moreover, we observed significant decreases in the redox status of ASC (ASC/TASC, ASC/DHA) and GSH (GSH/TGSH, GSH/GSSG), particularly in highly polluted sites (Supplementary Table S3). These results indicate that *S. portulacastrum* grown in Site 4 and 5 experienced oxidative stress.

Figure 4 depicts changes in the activity of the direct ROS scavenging enzymes POX, SOD, and CAT, as well as the ascorbate-glutathione cycle enzymes, in *S. portulacastrum* roots and shoots exposed to heavy metals. Activities were differentially enhanced in the shoots and roots of *S. portulacastrum* plants exposed to heavy metal treatments. Mainly in root tissues, POX, SOD and CAT showed remarkable increases in their activities (by 210, 47% and 120%, respectively) under heavy metal contamination in site 5 compared to their control values. When heavy metals reached the highest levels at site 5, such increases in POX, SOD, and CAT in shoots and roots were increased by 9%, and 9%, respectively. Furthermore, heavy metal stress significantly increased the activity of ASC/GSH recycling enzymes (APX, DHAR, MDHAR, GR, GPX) in both plant organs when compared to the corresponding controls (Figure 4). The highest heavy metal accumulation significantly decreased the level of ASC metabolizing enzymes (APX, DHAR, and MDHAR) and GSH metabolizing enzymes (GR, GPX) in both *S. portulacastrum* plants’ organs, but these activities did not significantly enhance in both *S. portulacastrum* organs compared to the corresponding as alone treatment.

### 3.6. Heavy Metal Detoxification Was More Pronounced in S. portulacastrum Roots

**Figure 5:**
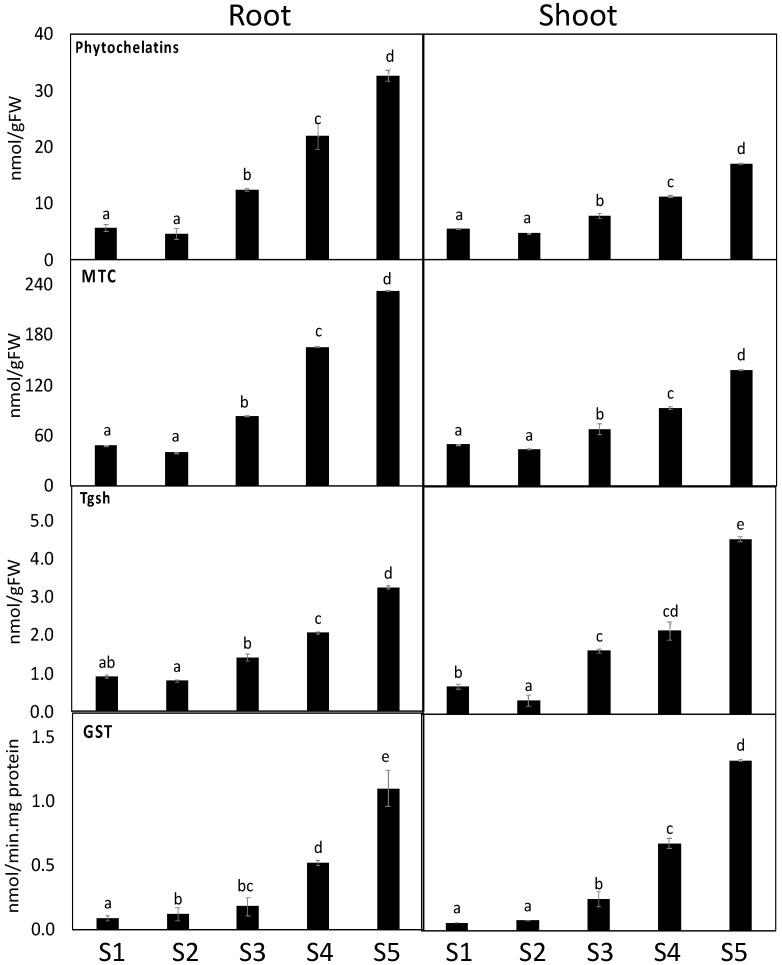
The impact of soil pollution detoxification-related metabolites and enzymes in the shoots and roots of *S. portulacastrum* grown in different control sites (site 5) and contaminated sites (site 1–4). Data are mean values ± SD (*n* = 4). Different letters indicate a statistically significant difference between means of the same plant species at significance level at least (*p* ≤ 0.05).

We measured metallothioneins (metal-binding proteins that regulate metal sequestration, MTC) and phytochelatins (gsh oligomers that sequester metals to the vacuole), total gsh, and glutathione-S-transferase (GST), which regulate glutathione–metal conjugation) to better understand heavy metal detoxification mechanisms in both plant organs (De Sousa et al., 2019). Heavy metal contamination increased the levels of Tgsh, and MTC and activity of GST in *S. portulacastrum* roots and shoots, but the level of phytochelatins was only increased in the root, compared to the corresponding control (Figure 5). On the other hand, heavy metal accumulation in Site 1 had no impact on levels of phytochelatins, Tgsh, and MTC, and the activity of GST as compared to the soil of controlled Site 1 (Figure 5). In addition, as compared to the shoot tissue of *S. portulacastrum* plants, root tissue shows higher levels of phytochelatins, Tgsh, MTC, and GST activity under heavy metal accumulation at sites 3 and 4.

### 3.7. Organ and Site-Specific Responses Are Supported by PCA Analysis

**Figure 6:**
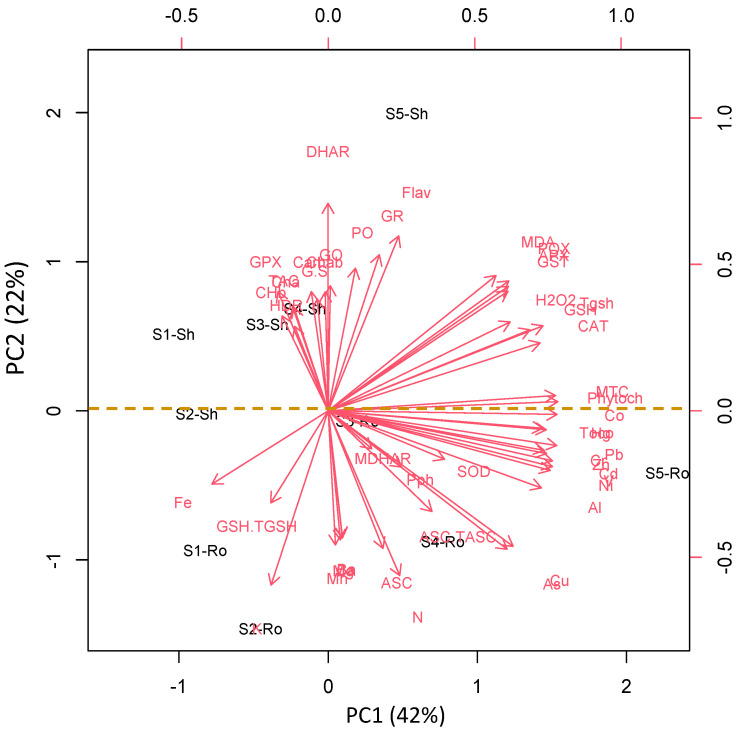
Principal component analysis (PCA) of parameters involved in photosynthesis, photorespiration, oxidative stress, detoxification, and antioxidant defense in the roots (Ro) and shoots (Sh) of *S. portulacastrum* grown in different control sites (S1) and contaminated sites (S2–5).

To test the specific responses of the roots and shoots of *S. portulacastrum* plant species to heavy metal stress, we performed a principal component analysis (PCA) with oxidative stress, antioxidant, and detoxification data set. The PCA embodied uniform metabolic/enzyme parameters along the first two dimensions (PC1 and PC2) that declared 48% and 17% of the data variability, respectively (Figure 6). PC1 separated the measured parameters based on sites’ induced oxidative and defensive responses (57% of all data variables), whereas the organ-specific responses were separated along PC2 (13% of all data variables). For control and low-stressed *S. portulacastrum* plants, PC1 showed that low heavy metal accumulation induced ASC level mainly in the root of Amaranthus plants grown in site 1 and site 5 and this effect was less pronounced in their shoot. Stress-related parameters such as photorespiration, antioxidants, detoxification, and oxidative stress markers were measured in the roots of highly stressed plants grown in Sites 2, 3, and 4, as well as stressed *S. portulacastrum* plants grown in site 4. PC2 showed organ specification in response to both control and heavy metal stress (Figure 6).

## 4. Discussion

### 4.1. Soil Contamination Impact on the Plant Cover and Biodiversity

Similar to the entire Khulais region, the studied area is an arid desert and predominated by therophytes [47,48,49]. Plants in the study area are adapted to environmental stressors, including water deficiency and extremely high temperatures. In addition to these factors, which are utilized to link species richness variability, soil pollution is another key element determining environmental variability. On the other hand, the change in distribution patterns is one of the key indicators of soil contamination [50] where species richness and diversity are known to be altered along a pollution gradient [5]. For example, species richness and composition were decreased in sites contaminated with heavy metals [51]. Here we also observed a decrease in species richness at the closest sites to the sewage lake (Table 1) that are mainly rich in stress tolerant species. This decrease in the plant cover indicates a decrease in soil health and fertility [52,53]. In the most contaminated site 5, there was a plant cover deterioration with a high drop in species number.

To evaluate the diversity and species richness, the Shannon index and Jaccard were applied. The index of diversity is a maximum when all species occur at the same relative frequency at a location to a minimum only when only one species is recorded [54]. Here, the highest diversity index was observed for the control sites (S1), while the contaminated site had values for the Shannon index and species richness due to the presence of a few species that were less susceptible to soil pollution (e.g., *Diptergium glaucum* and *Blepharis attenuate*) than other species. The Jaccard Index revealed changes in species richness between the control and contaminated sites, indicating a theoretical distribution of tolerant and sensitive species.

### 4.2. Identification and Selection of Tolerant Species

Compared to the control site, we also investigated the growth responses and biochemical mechanisms underpinning plants. Plants grown in heavy metal-enriched soils exhibited a slight reduction in their FW and DW (26% and 12% decrease, respectively). Our results correspond well with those of Yadav [1], who indicated the first visible sign of heavy metal toxicity is the reduction in the growth of plant shoots and roots. Several assumptions have been made to explain the adverse effects of heavy metals on plant growth, such as their interfering with essential micronutrients such as Mg [55]. Consequently, P deficiency affects vital physiological activities like the synthesis of ATP, glycolysis, respiration, and photosynthesis [56]. Furthermore, the observed reduction in the biomass can also be related to the inhibitory effect of heavy metals such as Pb, Co and Hg upon the photosynthetic efficiency. In this regard, heavy metals can interact with the photosynthetic machinery through their partition in leaf tissues, interact with key photosynthetic enzymes and alter chloroplast membranes. Excess heavy metals reduce photosystem II photosynthetic efficiency in cucumbers and beans, owing to decreased chlorophyll and carotenoids content [57,58]. Soil analysis for the content of heavy metals indicated the presence of several heavy metals with a high abundance of Pb, Co, Hg, and Cd. The accumulation of such heavy metals in agricultural land causes crop growth inhibition and productivity losses worldwide [13,59]. Therefore, only a few species colonized the contaminated sites close to the areas of the sewage, mainly included S. *portulacastrum* and *P. juliflora*. 

Similar to previous studies, these species showed tolerance to heavy metal pollution [60,61]. These tolerant species are not only pollution indicators but could also be used as bioremediators [62]. In this regard, bioremediation by higher plants is an efficient process that remediates soil pollutants [63]. Out of the identified tolerant species, we selected *S. portulacastrum* because it showed the highest relative densities and frequencies and at contaminated sites. We also investigated the growth responses and biochemical mechanisms underpinning stress tolerance in *S. portulacastrum* plants.

Similar to our study, the reduction in growth was in line with significant decreases (*p* < 0.05) in the levels of photosynthetic related parameters, i.e., Chl a and b in maize leaves grown in heavy metals contaminated soil [31]. These disturbances in the photosynthetic apparatus negatively impact several vital physiological and biochemical processes that impair plant growth [64]. For instance, the high heavy metals accumulation disturbed the electron transport chain and redox homeostasis, which led to increased ROS production [13,65,66,67]. Cu can also decrease the photosynthesis and stomatal conductance and the activity of Calvin cycle enzymes, especially glyceraldehyde-3-phosphate dehydrogenase and 3-phosphoglyceric acid kinase [68]. Similar to our study, the accumulation of H_2_O_2_ under heavy metal stress could be ascribed to the heavy metal-induced photorespiration-related parameters, an important H_2_O_2_ generating mechanism in plants, such as GOX and DHR enzymes [69].

Increases in ROS levels result in significant cell damage that leads to oxidative stress, including unspecific peroxidation of lipids, DNA, and proteins. Our results indicated a moderate increment in H_2_O_2_ levels, which was accompanied by an elevation in lipid peroxidation. Moreover, the observed decreases in the redox status of ASC and GSH, particularly in highly polluted sites suggesting *S. portulacastrum* experienced oxidative stress [10]. In agreement, Zhao et al. [70] reported heavy metals induced a remarkable increase in H_2_O_2_ levels in plants. Low accumulation of H_2_O_2_ in plants could play a signaling role in its resistance to heavy metal stress [65]. In this regard, low accumulation of H_2_O_2_ plays a protective mechanism through increased cell lignification to trap heavy metals [65].

Overall, under certain thresholds, low levels of ROS can initiate the synthesis of antioxidant scavenging enzymes, whereas high levels of ROS cause necrosis. Notably, changes in redox homeostasis in response to environmental cues, including heavy metal stress, may contribute to stress acclimation [71]. To scavenge H_2_O_2_ and to minimize the oxidative damage caused by environmental stress, antioxidant biosynthesis is induced as an adaptive response [31]. Increased levels of the antioxidant carotenoids in response to heavy metals play a significant role in photosystem protection. Antioxidants metabolites (like tocopherols, phenolics and flavonoids) as well as ROS-scavenging enzymes of the ASC-GSH cycle in addition to CAT, POX and SOD activities have been increased under metals stress [31,72]. Similar to our study, different organs showed different responses to ROS accumulation under heavy metal stress in shoots of *Solanum nigrum* showed quantitative and qualitative differences in antioxidant enzymes SOD, CAT activities and their isozymes expression from their roots. Non-enzymatic ROS-scavenging metabolites increase remarkably in heavy metals-treated plants in a species and organ-dependent manner [73,74,75]. For instance, phenolics metabolism showed a high increase in roots of Matricaria chamomilla under Cu stress while those in leaves did not change, indicating that their response is organ-specific [76].

### 4.3. S. portulacastrum Accumulated High Levels of Heavy Metals

Because heavy metals are toxic to plant growth and development [3,64], their removal from contaminated soil is critical. In this regard, many eco-friendly strategies use plants and their associated microbes to clean up soil pollutants. Therefore, we investigated the potential of the most tolerant species, *S. portulacastrum,* in bioremediation of different heavy metals. Heavy metal (e.g., Pb, Co and Hg) accumulation in the shoots and roots was measured. Then we calculated the bioconcentration (BCF) and translocation factor (TF). Stressed plant roots increase the excretion of organic acids in the soil that form chelates to modify the fixation and mobility of heavy metals in soils (Campbell and Nordstrom, 2014). Increasing citric acid release into the soil via root exudation, on the other hand, reduced heavy metal toxicity [77]. However, we observed a high value of the first factor (BCF) that was related to the metal accumulation in the root portion, indicating plant phytoextraction potential. Consistent with this, the plant demonstrated a low ability to phyto-stabilize, as evidenced by low heavy metal translocation to *S. portulacastrum* aerial tissues.

Plant root and shoot tissue induce the level of metal chelators (metallothioneins (MTC), phytochelatins (PCs), as well as the metal detoxification enzyme such as GST (regulates glutathione–metal conjugation) to overcome heavy metal toxicity [78]. In this regard, chelation of heavy metal ions with PCs is a key detoxification mechanism employed in several bioremediators [79]. Similar to the Gajewska et al. [80] study, we also observed high GST activity in response to heavy metal exposure. Similar to our results, plants exposed to Cd and As contamination experienced high levels in both MTC and PCs [15]. Therefore, our findings suggest that the enhanced detoxification system in *S. portulacastrum* could, in turn, promote plant growth under the challenge of heavy metal hazards.

In our study, the biochemical parameters that particularly increased in root or shoot can function as biomarker to select stress-tolerant plants for potential application for phytoremediation of areas highly polluted with heavy metals such as Cu, Ni, and As. In this regard, uses biochemical markers such as detoxification (MTC and PCs) and antioxidant (GSH, flavonoids, tocopherols) metabolites, and enzymes (GST, GR, POX, DHAR) activity to assess the influence of different soil heavy metals on plant growth and metabolism since these parameters are highly sensitive to the changes in the environment [18].

## 5. Conclusions

The effect of soil pollution in the Jeddah region, Saudi Arabia, on the distribution of plant species, their frequency, relative density, and vegetation cover, was studied. Furthermore, a stress-tolerant *S. portulacastrum* species was selected to evaluate bioremediation and stress defense strategies. The mechanistically based biochemical basis of *S. portulacastrum* living in contaminated soils, as well as its detoxification and antioxidant induction to alleviate oxidative stress of heavy metals, was identified and discussed. Our findings demonstrate the potential application of *S. portulacastrum* as a biomediator.

Therefore, the novelty of this work is that we introduced a promising heavy bio-accumulator and stress tolerance plant at the same time. We also uncovered its defense and detoxification mechanisms in its responses to complex heavy metal soil contaminations, to define key biochemical parameters as a potential biomarker in plants grown under heavy metal stress.

## 6. Future Perspectives and Recommendations

We recommend studying other plants that were registered in the study area, such as *Echinochloa colona*, *Prosopis juliflora* and *Leptochloa fusca* as phytoremediators.

## Figures and Tables

**Table 1:** Biodiversity indices for the studied sites and the relative density and frequency of *S. portulacastrum*. Different letters (a–e) represent the significant differences between the effect of heavy metal stress in the different target sites (Tukey test (*p* < 0.05)).

	S1	S2	S3	S4	S5
Evenness (R)	0.39 ^a^	0.36 ^a^	0.26 ^b^	0.24 ^b^	0.22 ^b^
Shannon Index	0.14 ^a^	0.16 ^a^	0.18 ^b^	0.19 ^c^	0.21 ^c^
Species richness	12 ^a^	10 ^b^	10 ^b^	9 ^c^	5 ^d^
Cover %	8 ^a^	9 ^a^	10 ^b^	10 ^b^	16 ^c^
*S. portulacastrum* density	0 ^a^	1.2 ^b^	7.3 ^c^	9.5 ^c^	10.33 ^d^
*S. portulacastrum* frequency	0 ^a^	15 ^b^	65 ^c^	83 ^d^	100 ^e^

**Table 2:** Concentration of heavy metals (µg/gm), minerals, phenols, acetic acid, pH, organic matter, electric conductivity and texture in the studied site’s soils. Different letters (a–d) represent the significant differences between the effect of heavy metal stress in the different target sites (Tukey test (*p* < 0.05)).

Location	S1	S2	S3	S4	S5
Cd (µg/gm)	0.43 ± 0.006 ^a^	1.456 ± 0.30 ^b^	3.045 ± 0.62 ^c^	4.962 ± 0.57 ^cd^	5.19 ± 0.37 ^d^
Ni (µg/gm)	0.441 ± 0.15 ^a^	0.403 ± 0.18 ^a^	0.804 ± 0.22 ^b^	1.115 ± 0.57 ^bc^	1.26 ± 0.71 ^c^
As (µg/gm)	0.461 ± 0.14 ^a^	0.336 ± 0.16 ^a^	0.232 ± 0.08 ^b^	0.350 ± 0.038 ^a^	0.45 ± 0.07 ^a^
Cu (µg/gm)	0.863 ± 0.02 ^a^	1.345 ± 0.14 ^b^	1.109 ± 0.17 ^a^	1.349 ± 1.05 ^b^	1.18 ± 0.14 ^a^
Pb (µg/gm)	0.072 ± 0.001 ^a^	1.154 ± 0.35 ^b^	4.734 ± 0.61 ^c^	7.408 ± 0.59 ^d^	10.70 ± 0.9 ^e^
Co (µg/gm)	0.108 ± 0.00 ^a^	0.751 ± 0.12 ^b^	3.917 ± 0.92 ^c^	5.767 ± 0.30 ^d^	8.67 ± 0.84 ^e^
Hg (µg/gm)	0.088 ± 0.01 ^a^	0.36 ± 0.01 ^b^	2.76 ± 0.603 ^c^	5.793 ± 0.45 ^d^	5.65 ± 0.70 ^d^
Al (µg/gm)	0.046 ± 0.02 ^a^	0.396 ± 0.17 ^b^	0.323 ± 0.36 ^b^	0.629 ± 0.05 ^c^	0.72 ± 0.21 ^c^
V (µg/gm)	0.046 ± 0.004 ^a^	0.315 ± 0.13 ^b^	0.470 ± 0.14 ^b^	0.682 ± 0.63 ^c^	0.82 ± 0.17 ^d^
Cr (µg/gm)	0.060 ± 0.02 ^a^	0.261 ± 0.06 ^b^	0.617 ± 0.16 ^c^	0.735 ± 0.00 ^c^	1.02 ± 0.24 ^d^
Zn (µg/gm)	0.046 ± 0.0 ^a^	0.096 ± 0.01 ^b^	0.594 ± 0.05 ^c^	0.776 ± 0.097 ^c^	0.89 ± 0.08 ^d^
Mn (µg/gm)	0.079 ± 0.01 ^a^	0.15 ± 0.02 ^b^	0.072 ± 0.01 ^a^	0.178 ± 0.08 ^b^	0.08 ± 0.010 ^b^
Mg (µg/gm)	0.064 ± 0.007 ^a^	0.142 ± 0.02 ^b^	0.062 ± 0.00 ^a^	0.159 ± 0.07 ^b^	0.07 ± 0.01 ^a^
Ca (µg/gm)	0.050 ± 0.017 ^a^	0.104 ± 0.01 ^b^	0.047 ± 0.00 ^a^	0.117 ± 0.04 ^b^	0.05 ± 0.00 ^a^
Ba (µg/gm)	0.028 ± 0.01 ^a^	0.069 ± 0.02 ^b^	0.029 ± 0.01 ^a^	0.076 ± 0.02 ^b^	0.03 ± 0.01 ^a^
Fe (µg/gm)	0.565 ± 0.08 ^c^	0.134 ± 0.03 ^b^	0.060 ± 0.01 ^a^	0.151 ± 0.07 ^b^	0.06 ± 0.01 ^a^
K (µg/gm)	1.422 ± 0.13 ^c^	1.06 ± 0.00^ab^	0.871 ± 0.12 ^a^	1.100 ± 0.8 ^ab^	0.87 ± 0.07 ^a^
N (µg/gm)	8.56 ± 1.3 ^b^	7.73 ± 2.17 ^a^	7.68 ± 0.99 ^a^	8.14 ± 7.9 ^ab^	7.77 ± 0.3 ^a^
Phenols (µg/gm)	32.15 ± 0.08 ^bc^	12.63 ± 3.0 ^a^	27.02 ± 4.2 ^b^	43.39 ± 3.7 ^d^	59.1 ± 5.7 ^d^
Citric acid (µg/gm)	31.50 ± 3.8 ^c^	4.86 ± 0.86 ^a^	5.26 ± 1.09 ^a^	5.75 ± 0.1 ^a^	7.20 ± 0.64 ^b^
pH	7.3 ± 0.01 ^a^	7.3 ± 0.01 ^a^	7.3 ± 0.03 ^a^	7.4 ± 0.02 ^a^	7.80 ± 0.09 ^a^
O.M (%)	0.72 ± 0.09 ^a^	1.38 ± 0.03 ^a^	1.41 ± 0.02 ^a^	1.61 ± 0.02 ^a^	1.94 ± 0.03 ^a^
E.C (ds m^−1^)	0.71 ± 0.01 ^b^	0.91 ± 0.01 ^b^	0.85 ± 0.01 ^b^	0.99 ± 0.01 ^a^	1.05 ± 0.01 ^a^
Sands (%)	61.09 ± 1.30 ^a^	67.74 ± 3.20 ^a^	61.49 ± 1.60 ^a^	70.78 ± 1.36 ^a^	73.99 ± 0.04 ^a^
Silts (%)	21.6 ± 0.89 ^a^	17.90 ± 1.04 ^a^	21.2 ± 0.89 ^a^	16.00 ± 0.68 ^a^	14.44 ± 0.04 ^a^
Clay (%)	17.31 ± 0.78 ^a^	14.36 ± 1.05 ^a^	17.31 ± 0.8/8 ^a^	13.22 ± 0.74 ^a^	11.57 ± 0.7 ^a^

**Table 3:** Heavy metal concentrations (µg/gm) in the shoots and roots of *S. portulacastrum* grown in control site (site 1) and contaminated sites (site 2–5). Data are mean values ± SE (*n* = 4). Different letters (a–e) represent the significant differences between the effect of heavy metal stress in the different target sites (Tukey test (*p* < 0.05)).

	S1		S2		S3		S4		S5	
	Shoot	Root	Shoot	Root	Shoot	Root	Shoot	Root	Shoot	Root
Cd	1.90 ±0.01 ^a^	13.39 ± 2.74 ^a^	21.27 ± 5.1 ^b^	57.52 ± 6.5 ^b^	46.5 ± 7.3 ^c^	127.9 ± 13 ^c^	76.8 ± 6.5 ^d^	202.4 ± 22 ^d^	73.21 ± 8.6 ^e^	296.63± 64 ^e^
Ni	1.94 ± 0.35 ^a^	25.18 ± 9.3 ^a^	11.44 ± 2.68 ^b^	30.97 ± 3.4 ^b^	24.5 ± 1.27 ^c^	68.8 ± 12.5 ^c^	35. ± 3.4 ^d^	99.4 ± 14.5 ^d^	40.9 ± 4.4 ^e^	134.9 ± 27.1 ^e^
As	2.03 ± 0.3 ^a^	24.64 ± 4.6 ^a^	9.54 ± 2.3 ^bc^	25.78 ± 2.9 ^a^	7.20 ± 0.4 ^b^	20.19 ± 3.5 ^a^	10.54 ± 0.3 ^c^	29.70 ± 5.9 ^b^	13.2 ± 0.24 ^d^	44.50 ± 10.8 ^c^
Cu	3.80 ± 0.06 ^a^	47.48 ± 11 ^a^	38.19 ± 2.1 ^b^	107.16 ± 19 ^b^	32.4 ± 1.8 ^b^	91.1 ± 16.2 ^b^	38.7 ± 1.1 ^b^	109.2 ± 21 ^b^	33.33 ± 2.0 ^b^	166.84 ± 48 ^c^
Pb	0.32 ± 0.01 ^a^	1.81 ± 0.57 ^a^	11.34 ± 3.2 ^b^	57.05 ± 10.0 ^b^	47.4 ± 7.0 ^b^	138. ± 50.7 ^c^	77.0 ± 11 ^d^	258.2 ± 59 ^d^	121 ± 8.8 ^e^	496.69 ± 63 ^e^
Co	0.47 ± 0.00 ^a^	2.75 ± 0.9 ^a^	7.72 ± 0.89 ^b^	22.38 ± 7.4 ^b^	39.6 ± 3.03 ^c^	114.2 ± 34 ^c^	60.1 ± 1.0 ^d^	177.7 ± 36 ^d^	159.17 ± 43 ^c^	490.17 ± 71 ^e^
Hg	0.40 ± 0.03 ^a^	2.30 ± 0.7 ^a^	3.83 ± 0.75 ^b^	16.14 ± 1.4 ^b^	28.74 ± 4.3 ^c^	47.65 ± 4.9 ^c^	63.0±13.1 ^d^	186. ± 38.3 ^d^	81.60 ± 5.1 ^e^	205.21 ± 45 ^e^
Al	0.19 ± 0.03 ^a^	1.03 ± 0.25 ^a^	3.91 ± 0.09 ^b^	11.05 ± 2.2 ^c^	2.65 ± 1.23 ^c^	6.84 ± 2.2 ^b^	5.2 ± 1.03 ^d^	15.32 ± 6.3 ^d^	7.08 ± 0.37 ^e^	24.05 ± 6.8 ^e^
V	0.20 ± 0.01 ^a^	1.16 ± 0.36 ^a^	3.14 ± 0.08 ^b^	8.85 ± 1.82 ^b^	4.79 ± 0.20 ^c^	13.69 ± 3.6 ^c^	6.76 ± 1.4^d^	19.97 ± 8.5 ^d^	8.78 ± 1.08^e^	30.22 ± 10.0 ^e^
Cr	0.26 ± 0.05 ^a^	1.63 ± 0.7 ^a^	2.62 ± 0.20 ^b^	7.53 ± 2.2 ^b^	6.92 ± 1.62 ^c^	27.93 ± 3.0 ^c^	8.32 ± 1.8 ^c^	24.6 ± 10.8 ^c^	11.39 ± 2.7 ^d^	40.07 ± 16 ^d^
Zn	0.20 ± 0.01 ^a^	1.46 ± 0.31 ^a^	1.01 ± 0.24 ^a^	3.60 ± 0.7 ^b^	6.23 ± 1.4 ^b^	22.33 ± 4.5 ^c^	8.1 ± 1.9b ^c^	29.1 ± 5.9 ^cd^	9.32 ± 2.22 ^c^	39.65 ± 9.4 ^d^
Mn	2.22 ± 0.08 ^a^	3.00 ± 0.32 ^a^	4.58 ± 0.23 ^b^	6.18 ± 0.75 ^b^	2.07 ± 0.09 ^a^	2.79 ± 0.31 ^a^	5.1 ± 0.26 ^b^	6.97 ± 0.80^b^	2.19 ± 0.11 ^a^	2.96 ± 0.34 ^a^
Mg	1.77 ± 0.08 ^a^	2.39 ± 0.27 ^a^	4.14 ± 0.26 ^b^	5.59 ± 0.68 ^b^	1.79 ± 0.10 ^a^	2.41 ± 0.28 ^a^	4.6 ± 0.28 ^b^	6.21 ± 0.75 ^b^	1.94 ± 0.12 ^a^	2.62 ± 0.32 ^a^
Ca	1.29 ± 0.11 ^a^	1.73 ± 0.23 ^a^	3.08 ± 0.06 ^b^	4.16 ± 0.43 ^b^	1.32 ± 0.01 ^a^	1.78 ± 0.18 ^a^	3.4 ± 0.04 ^b^	4.62 ± 0.47 ^b^	1.46 ± 0.01 ^a^	1.96 ± 0.20 ^a^
Ba	0.88 ± 0.09 ^a^	1.19 ± 0.17 ^a^	2.16 ± 0.14 ^b^	2.91 ± 0.36 ^b^	0.92 ± 0.01 ^a^	1.25 ± 0.13 ^a^	2.4 ± 0.13 ^b^	3.24 ± 0.38 ^b^	1.01 ± 0.04 ^a^	1.36 ± 0.15 ^a^
Fe	15.32 ± 0.0 _a_	20.67 ± 2.1 ^a^	3.98 ± 0.4 ^b^	5.36 ± 0.8 ^b^	1.74 ± 0.15 ^c^	2.35 ± 0.30 ^c^	4.4 ± 0.4 ^b^	5.98 ± 0.8 ^b^	1.87 ± 0.18 ^c^	2.53 ± 0.30 ^c^
K	39 ± 0.70 ^a^	53.10 ± 5.5 ^a^	30.32 ± 0.1 ^b^	40.90 ± 4.1 ^b^	25.62 ± 0.8 ^c^	34.57± 3.70 ^c^	31 ± 0.52 ^b^	42.39 ± 4.3 ^b^	24.2 ± 0.22 ^c^	32.68 ± 3.34 ^c^
N	244.6 ± 18 ^a^	330 ± 42 ^a^	204.8 ± 16.9 ^b^	276.35 ± 37 ^b^	225.8 ± 5 ^b^	304.6 ± 30 ^ab^	234 ± 5.2 ^ab^	316.9 ± 33 ^ab^	217± 0.46 ^c^	293.6 ± 29 ^ab^

**Table 4:** Biological concentration factor (BCF) and translocation factor (TF) of *S. portulacastrum* grown in different control sites (Site 1) and contaminated sites (Sites 2–5). Data are mean values ± SE (*n* = 4). Different letters (a–c) represent the significant differences between the effect of heavy metal stress in the different target sites (Tukey test (*p* < 0.05)).

	S1		S2		S3		S4		S5	
	BCF	TF	BCF	TF	BCF	TF	BCF	TF	BCF	TF
Cd	30.98 ± 2.10 ^a^	0.14 ± 0.09 ^a^	39.51 ±1.05 ^b^	0.4 ± 0.02 ^c^	42.0 ± 2.3 ^c^	0.36 ± 0.02 ^c^	40.8 ± 1.0 ^c^	0.38 ± 0.02 ^c^	57.20 ± 2.1 ^d^	0.25 ± 0.05 ^b^
Ni	57.05 ± 2.5 ^a^	0.08 ± 0.01 ^a^	76.92 ± 2.3 ^b^	0.37 ±0.13 ^b^	85.66± 2.7 ^c^	0.36 ± 0.05 ^b^	89.18 ± 2.3 ^c^	0.36 ± 0.01 ^b^	107.11 ±14 ^d^	0.30 ± 0.08 ^b^
As	53.44 ± 1.3 ^a^	0.08 ± 0.02 ^a^	76.80 ± 2.4 ^b^	0.36 ± 0.1 ^b^	87.1 ± 3.1 ^b^	0.35 ± 0.04 ^b^	84.95 ± 1.5 ^b^	0.35 ± 0.05 ^b^	99.56 ± 11.3 ^c^	0.29 ± 0.04 ^c^
Cu	55.00 ± 2.6 ^a^	0.08 ± 0.01 ^a^	79.69 ± 2.4 ^b^	0.36 ± 0.12 ^c^	82.12 ± 2.5 ^c^	0.36 ± 0.05 ^c^	80.95 ± 1.2 ^c^	0.35 ± 0.01 ^c^	141.31 ± 18 ^d^	0.20 ± 0.02 ^b^
Pb	25.20 ± 1.03 ^a^	0.17 ± 0.03 ^a^	49.45 ± 2.3 ^c^	0.2 ± 0. 0 ^a^	29.2 ± 1.2 ^a^	0.34 ± 0.0 ^b^	34.8 ± 1.02 ^b^	0.30 ± 0.01 ^b^	46.43 ± 2.3 ^c^	0.24 ± 0.01 ^a^
Co	25.58 ± 1.5 ^a^	0.17 ± 0.03 ^a^	29.81 ± 1.02 ^a^	0.3 ± 0.08 ^b^	29.1 ± 1.3 ^b^	0.35 ± 0.05 ^b^	30.8 ± 1.0 ^b^	0.34 ± 0.06 ^b^	56.51 ±11.3 ^c^	0.32 ± 0.02 ^b^
Hg	26.03 ± 1.6 ^b^	0.17 ± 0.01 ^a^	43.98 ± 1.2 ^d^	0.24 ± 0.0 ^a^	17.22 ± 0.9 ^a^	0.60 ± 0.08 ^d^	32.1 ± 1.04 ^b^	0.34 ± 0.02 ^b^	36.33 ± 3.6 ^c^	0.40 ± 0.04 c
Al	22.44 ± 1.4 ^a^	0.18 ± 0.01 ^a^	27.87 ± 1.4 ^c^	0.35 ± 0.0 ^c^	21.16 ± 1.5 ^a^	0.39 ± 0.04 ^c^	24.3 ± 1.08 ^b^	0.34 ± 0.0 ^bc^	33.51 ± 2.5 ^c^	0.29 ± 0.01 ^b^
V	25.18 ± 1.5 ^a^	0.17 ± 0.02 ^a^	28.13 ± 2.0 ^a^	0.35 ± 0.08 ^b^	29.10 ± 1.0 ^a^	0.35 ± 0.03 ^b^	29.2 ± 1.02 ^a^	0.34 ± 0.07 ^b^	36.80 ± 2.1 ^b^	0.29 ± 0.03 ^b^
Cr	27.26 ± 1.0 ^a^	0.16 ± 0.00 ^a^	28.87 ± 1.09 ^a^	0.35 ± 0.1 ^c^	45.27 ± 1.5 ^c^	0.25 ± 0.03 ^b^	33.4 ± 0.3 ^b^	0.34 ± 0.1 ^c^	39.15 ± 3.6 ^b^	0.28 ± 0.01 ^b^
Zn	31.80 ± 1.3 ^a^	0.14 ± 0.03 ^a^	37.68 ± 1.5 ^ab^	0.28 ± 0.03 ^c^	37.6 ± 1.0 ^ab^	0.28 ± 0.0 ^c^	37.6 ± 0.8 ^ab^	0.28 ± 0.02 ^b^	44.60 ± 2.50 ^c^	0.24 ± 0.01 ^b^
Fe	36.59 ± 2.1 ^a^	0.74 ± 0.02 ^a^	39.93 ± 1.04 ^b^	0.74 ± 0.6 ^a^	39.12 ± 1 ^b^	0.74 ± 0.14 ^a^	39.6 ± 0.25 ^b^	0.74 ± 0.14 ^a^	39.51 ± 1.5 ^b^	0.74 ± 0.01 ^a^

**Table 5:** Fresh weights, dry weights and growth pigments of *S. portulacastrum* grown in different control sites (Site 1) and contaminated sites (Site 2–5). Data are mean values ± SE (*n* = 4). Different letters (a–d) represent the significant differences between the effect of heavy metal stress in the different target sites (Tukey test (*p* < 0.05)).

	S1		S2		S3		S4		S5	
	Root	Shoot	Root	Shoot	Root	Shoot	Root	Shoot	Root	Shoot
FW gm	--	--	84.21 ±5.3 ^a^	385 ± 12.30 ^a^	78.25 ± 3.4 ^a^	315 ± 10.90 ^b^	56.24 ± 4.2 ^d^	294 ± 14.20 ^d^	42.36 ± 3.1 ^a^	285 ± 11.07 ^c^
DW gm	--	--	16.25 ±1.3 ^b^	89.5 ± 6.05 ^b^	13.25 ± 1.2 ^a^	82.8 ± 5.62 ^a^	11.35 ± 1 ^a^	79.3 ± 5.4 ^a^	10.58 ± 0.6 ^d^	77.2 ± 4.81 ^c^
CH a		2.67 ± 0.2 ^ab^		2.62 ± 0.3 ^ab^		2.01 ± 0.04 ^a^		3.4 ± 0.23 ^c^		3.23 ± 0.6 ^c^
CH b		0.66 ± 0.02 ^a^		0.74 ± 0.1 ^b^		0.59 ± 0.06 ^a^		0.66 ± 0.05 ^a^		0.73 ± 0.0 ^b^
Ch a + Ch b		1.51 ± 1.3 ^a^		1.51 ± 1.3 ^a^		1.51 ± 1.3 ^a^		3.47 ± 0.2 ^b^		3.23 ± 0.6 ^b^
Carotenoids		24.23 ± 0.6 ^a^		24.1 ± 0.31 ^a^		33.06 ± 1.0 ^b^		36.40 ± 4.3 ^b^		42.95 ± 3.1 ^c^

**Table 6:** Oxidative stress markers of *S. portulacastrum* grown in different control sites (Site 1) and contaminated sites (Site 2–5). Data are mean values ± SE (*n* = 4). Different letters (a–d) represent the significant differences between the effect of heavy metal stress in the different target sites (Tukey test (*p* < 0.05)).

	S1		S2		S3		S4		S5	
	Root	Shoot	Root	Shoot	Root	Shoot	Root	Shoot	Root	Shoot
GO		0.70 ± 0.04 ^a^		0.78 ± 0.02 ^a^		0.90 ± 0.0 ^ab^		0.85 ± 0.1 ^ab^		1.60 ± 0.04 ^b^
G/S ratio		21.46 ± 4.28 ^a^		20.94 ± 4.06 ^a^		19.09 ± 0.2 ^a^		26.3 ± 2.00 ^b^		35.29 ± 3.06 ^b^
HDR		1.80 ± 0.23 ^a^		2.01 ± 0.45 ^a^		2.20 ± 0.00 ^a^		1.83 ± 0.11 ^a^		3.69 ± 0.69 ^b^
H_2_O_2_	217.2 ± 53.0 ^a^	235.0 ± 8.5 ^a^	241.5± 3.3 ^ab^	254.2 ± 14.2 ^b^	282.4 ± 14.4 ^b^	333.0 ± 51.5 ^c^	305.79 ± 8.9 ^b^	307.6 ± 9.7 ^bc^	354.3 ± 18.4 ^c^	314 ± 16.6 ^bc^
MDA	2.31 ± 0.55 ^a^	2.88 ± 0.23 ^a^	3.00 ± 0.06 ^b^	3.36 ± 0.13 ^b^	4.69 ± 0.14 ^a^	3.72 ± 0.45 ^b^	5.13 ± 0.34 ^c^	5.01 ± 0.00 ^c^	5.54 ± 0.17 ^c^	7.28 ± 0.94 ^d^
PO	1.44 ± 0.01 ^a^	1.74 ± 0.04 ^a^	1.50 ± 0.07 ^a^	1.39 ± 0.18 ^a^	1.93 ± 0.16 ^ab^	1.63 ± 0.10 ^a^	1.42 ± 0.10 ^a^	1.52 ± 0.04 ^a^	1.56 ± 0.04 ^a^	2.88 ± 0.03 ^c^

## Data Availability

Data is contained within the article or supplementary material.

## Supplementary Materials

The following are available online at www.mdpi.com/2076-3921/11/1/19/s1, Table S1: Monthly variations of rainfall, relative humidity in the study area. Table S2: List of the recorded species with their, families, relative densities (RD) and frequencies (F) in the different sites. Table S3: Redox status of ASC (ASC/TASC, ASC/DHA) and GSH (GSH/TGSH, GSH/GSSG) of *S. portulacastrum* grown in different control site (Site 1) and contaminated sites (Site 2–5).
