# Supplementary material for: High Redox Status as the Basis for Heavy Metal Tolerance of Sesuvium portulacastrum L. Inhabiting Contaminated Soil in Jeddah, Saudi Arabia
Source: Antioxidants (Basel). 2021 Dec 22;11(1):19. doi: 10.3390/antiox11010019 (PMC8773048; doi:10.3390/antiox11010019)
Supplement: Supplementary file 1 [file antioxidants-11-00019-s001.zip › antioxidants-1510203-supplementary.pdf]

Table S1: Monthly variations of rainfall, relative humidity in the study area. The data were extracted from the Jeddah meteorological station (Saudi Arabia).

	**Temperature ºC**			**Relative Humidity (%)**			**Precipitation (ml)**
	**Mx**	**Mn**	**M**	**Mx**	**Mn**	**M**	
January	26.8	18.2	22.3	81	8	46	0
February	28	19.6	23.6	87	14	53	1
March	30.5	20.7	25.3	95	9	55	6.2
April	32.8	23.2	27.8	83	17	53	0
May	35.9	26.8	31.2	84	8	49	0
June	37.9	27.3	32.3	86	5	52	0
July	38.5	29.1	33.6	93	12	49	2
August	38.7	30	34.2	81	19	52	0
September	38.7	30	33.9	88	5	58	TRACE
October	39.2	27	32.4	89	3	49	3.8
November	33.1	25.3	28.8	88	23	57	19
December	31.9	22.4	26.6	84	8	53	8

Table S2: List of the recorded species with their, families, relative densities (RD) and frequencies (F) in the different sites.

families	Species	S1		S2		S3		S4		S5	
		RD	F	RD	F	RD	F	RD	F	RD	F
Aizoaceae	*Blepharis attenuate* Napper	2.7	15.6	0	0	0	0	0	0	0	0
	Aizoon canariense L.	1.3	8	0	0	0	0	0	0	0	0
Aizoaceae	*Sesuvium portulacastrum* L.	0	0	1.2	15	7.3	65	9.5	83	10.3	100
	Trianthema portulacastrum L.	1	0	6.7	25	3	8.6	2	13	0	0
Asclepiedaceae	*Calotropis procera* (Aiton) W.T.Aiton	1.9	15	1.2	3	0	0	0	0	0	0
Boraginaceae	*Heliotropium bacciferum* Forssk.	0	0	1	5	1.2	13	1	11	0	0
	Mareua oblongifolia (Frossk.) A.Rich.	1.5	6	0	0	0	0	0	0	0	0
Capparaceae	*Diptergium glaucum* Decne.	1.3	11	0	0	0	0	0	0	0	0
	Cadaba glandulosa Frossk.	0	0	0	0	0	0	1.1	0	0	0
Chenopodiacea	*Salsola tetrandra* Forssk	0	0	1	7	1.1	9	1	9	0	0
	Suaeda monica Frossk.ex J.F. Gmel.	1.5	12	1.3	21	0	0	0	0	0	0
	Acacia hamulosa Benth.	4.3	27	0	0	0	0	0	0	0	0
Fabaceae	*Acacia tortilis* (Forssk.) Hayne	3.2	16	0	0	0	0	0	0	0	0
	Prosopis Juliflora (Sw.) DC.	0	0	1.5	11	1.9	12	2.4	85	3.5	90
Malvaceae	*Abutilon pannosum* (G. Forst.) Schltdl.	1.1	3	0	0	0	0	0	0	0	0
	Cynodon dactylon (L.) Pers.	1.5	7	0	0	0	0	0	0	0	0
	Echinochloa colona (L.) Link	0	0	7.4	45	6.8	24	8.9	50	0	0
Poaceae	*Leptochloa fusca* (L.) Kunth	0	0	0	0	5.7	19	1	4	9.1	60
	Phragmites australis (Cav.)Trin ex Steud	0	0	0	0	2.3	14	21	63	11	14
	Stipa capensis Thunb.	1.3	12	0	0	0	0	0	0	0	0
Portulacaeae	*Portulaca oleraceae* L.	0	0	6.2	14	5.9	19	0	0	0	0
Solanaceae	*Solanum incanum* L.	3.4	16	0	0	0	0	0	0	0	0
Tamaricaceae	*Tamarix nilotica* (Ehrenb.) Bunge	0	0	0	0	2.1	13	1	3	1.1	12
Zygophyllaceae	*Fagonia mollis* Delile.	2.7	5	0	0	0	0	0	0	0	0

Table S3: Redox status of ASC (ASC/TASC, ASC/DHA) and GSH (GSH/TGSH, GSH/GSSG) of *S. portulacastrum* grown in different control site (Site 1) and contaminated sites (Site 2-5). Data are mean values ± SE (n=4). Different letters represents the significant differences between the effect of heavy metal stress in the different target sites (Tuckey test (P < 0.05)).

	**S1**		**S2**		**S3**		**S4**		**S5**	
	**Root**	**Shoot**	**Root**	**Shoot**	**Root**	**Shoot**	**Root**	**Shoot**	**Root**	**Shoot**
ASC/TASC	67.3±3.9^{a}	54.6±0.3^{a}	53.9±0.2^{ab}	41.6±2.5^{b}	43.2±0.4^{b}	30±1.4^{c}	42.3±9.1^{ab}	31±0.5^{d}	33.3±4.1^{c}	27.9±0.1^{d}
ASCH/DHA	0.86±0.04^{a}	0.91±0.07^{a}	0.69±0.05^{b}	0.73±0.1^{b}	0.42±0^{c}	0.53±0.03^{c}	0.34±0^{d}	0.39±0.02^{d}	0.24±0.09^{e}	0.32±0.01^{d}
GSH/TGSH	50.9±1.9^{a}	47.6±2.9^{a}	40.2±2.1^{ab}	26.9±1.6^{b}	15.9±1.3^{c}	25.5±0.4^{b}	24.9±0.3^{b}	22±0.6^{b}	17.3±1.6^{c}	13.3±1.7^{c}
GSH/GSSG	0.53±0.07^{a}	0.44±0.07^{a}	0.25±0.04^{b}	0.34±0.09^{b}	0.21±0.01^{b}	0.34±0.07^{b}	0.262±0.06^{b}	0.36±0.02^{b}	0.162±0.1^{c}	0.18±0.05c
